# Supplementary material for: Maternal mental health and nutritional status of infants aged under 6 months: A secondary analysis of a cross-sectional survey
Source: PLOS Glob Public Health. 2024 Sep 13;4(9):e0003139. doi: 10.1371/journal.pgph.0003139 (PMC11398659; doi:10.1371/journal.pgph.0003139)
Supplement: S1 Checklist — (DOCX) [file pgph.0003139.s001.docx]

Inclusivity in global research

PLOS’ policy on inclusivity in global research aims to improve transparency in the reporting of research performed outside of researchers’ own country or community and ensures that PLOS publications reporting global research adhere to high standards for research ethics and authorship. Authors of relevant research articles may be asked to complete the questionnaire below, which outlines ethical, cultural, and scientific considerations specific to inclusivity in global research. This questionnaire may be requested when researchers have travelled to a different country to conduct research, if research uses samples collected in another country, research with Indigenous populations or their lands, or if research is on cultural artefacts. Researchers travelling to another country solely to use laboratory equipment will not normally be required to complete the questionnaire. However, the questionnaire can be requested at the journal’s discretion for any submission – if you have been requested to complete this questionnaire by the PLOS journal you submitted to, please do so.

Please complete the questionnaire below and include this as a Supporting Information file with your manuscript. Note that if your paper is accepted for publication, this checklist will be published with your article in the supporting information files. Please ensure that you reference the checklist in the main body of your manuscript. We suggest adding a subsection ‘Inclusivity in global research’ to your Methods section and adding the following sentence: “Additional information regarding the ethical, cultural, and scientific considerations specific to inclusivity in global research is included in the Supporting Information (SX Checklist)”

The questions have been designed to be applicable to a wide range of study types, and there are subsections for both human subjects research and non-human subjects research. If any of the questions are not relevant to your research please mark them as “N/A” as appropriate.

**Ethical considerations, permits and authorship**

*This section is applicable to all research types.*

Provide details as to who granted permissions and/or consent for the study to take place in the Methods section of your manuscript. This should include the names of **all** ethics boards, governmental organizations, community leaders or other bodies that provided approval for the study. If individuals provided approval refer to these people by their role or title but do not list their name(s).

We had ethical permission from Jimma University and London School of Hygiene & Tropical Medicine Ethics committees. (please see details on page number 6)

Prior to the work starting we also got permission from local zonal health bureau.

If there were any deviations from the study protocol after approval was obtained please provide details of these changes in the Methods section of your manuscript.
Did this study involve local collaborators that are residents of the country where the research was conducted or members of the community studied? If you do not have any authors from said communities, please provide an explanation for this below.

This paper arose from a Jimma University Ethiopia/Goal Ethiopia/ENN Oxford/LSHTM UK collaboration. All are represented in the author team. Ethiopian authors led the fieldwork and also led this paper as 1^st^ author (Jimma University).

Reported on page number: There were no deviations from this protocol

Everyone listed as an author should meet PLOS’ criteria for authorship and all individuals who meet these criteria should be included in the author byline, rather than the acknowledgements. For further information please see the journal’s Authorship Policy.

**Human subjects research (e.g. health research, medical research, cross-cultural psychology)**

Did you obtain written informed consent from a representative of the local community or region before the research took place? How did you establish who speaks for the community? Details of written informed consent obtained from study participants should be reported separately in the Methods section of your manuscript.

Yes, ethical approval for the original data was granted by Jimma University Institutional Review Board (ref: IHRPGD/478//2020) and by the Research Ethics Committee at the London School of Hygiene and Tropical Medicine (ref 18022). Informed consent/permission was granted by the local Zonal health bureau. Written informed consent was obtained from the mother/caregivers (guardians) of every infant for participation by trained research nurses. Indicated on page 6.

How did members of the local community provide input on the aims of the research investigation, its methodology, and its anticipated outcome(s)?

This paper and a related qualitative paper arose from formative work done towards a large cluster RCT (<https://www.isrctn.com/ISRCTN47300347> ) and are designed to get exactly this local community input on the wider research on infant malnutrition.

Even at the development phase of the formative survey which gave rise to this piece, we consulted the local community, potential stakeholders and researchers on the area. It also builds on many years stakeholder engagement and other work in this area through the MAMI Global Network (<https://www.ennonline.net/ourwork/research/mami>). We considered comments and feedback in the protocol and the research tool before the data collection.

When engaging with the local community, how did you ensure that the informed consent documents and other materials could be understood by local stakeholders?

Our community engagement strategy has taken into account the educational background, local language and other contexts that could influence understanding related to our research conduct such on information sheet, consent sheet and other related documents. Thus we translated these documents into the local languages (Oromifaa and Amharic) and study communications were also done in local languages. In the case of illiterate participants, we used standard ethics-committee-approved witness procedures involving a family/community member who is literate and can vouch for the validity of the procedure.

Will the findings of the research be made available in an understandable format to stakeholders in the community where the study was conducted (e.g. via a presentation, summary report, copies of publications, etc.)? Please provide details of how this will be achieved.

We are doing a series of dissemination events for our main project, which include meetings for national, Zonal and local community level stakeholders, each customized to their level for better understanding and engagement.

**Non-human subjects research using specimens/ animals collected as part of the study, or those housed in archival collections. Examples include archaeology, paleontology, botany and zoology.**

Did the permission you obtained from a local authority to perform the study include an agreement on access to outputs and benefit sharing? This may include procedures to enable fair distribution of the benefits and resources arising from the research performed. Please include any details of Prior Informed Consent and Benefit Sharing Agreements obtained. These may be required by field-specific regulations, for example the Convention on Biological Diversity (CBD) and the associated Nagoya Protocol.

This study does not involve any of the above. So this section is not applicable (N/A) to this research.

If the material used in your study was imported, please A) provide the year it was imported and B) indicate whether permits were obtained to import/export the materials used, C) provide details of any permits obtained. If this information is not available, please indicate this.

N/A

If you used archival specimens, please state how the material used in your study was acquired by the institute it is held in and provide details of any permits obtained for the original excavations/ sample collection. If this information is not available, please indicate this.

N/A

How was the potential cultural significance of the materials collected in your study to local communities considered in your research design? Were Indigenous peoples and/or local researchers and institutions involved with archaeological excavations / collection of specimens? If so, please provide a description of their involvement.

N/A

If your manuscript includes photographs of human remains please indicate whether authors obtained permission from descendants or affiliated cultural communities to do so.

N/A
