# Supplementary material for: Maternal mental health and nutritional status of infants aged under 6 months: A secondary analysis of a cross-sectional survey
Source: PLOS Glob Public Health. 2024 Sep 13;4(9):e0003139. doi: 10.1371/journal.pgph.0003139 (PMC11398659; doi:10.1371/journal.pgph.0003139)
Supplement: Attachment — Submitted filename: PGPH-D-24-00706_reviewer_R1.pdf [file pgph.0003139.s002.pdf]

PLOS Global Public Health

Maternal mental health and nutritional status of infants aged under 6 months: a secondary analysis of a cross-sectional survey

--Manuscript Draft--

**Manuscript Number:**	PGPH-D-24-00706
**Article Type:**	Research Article
**Full Title:**	Maternal mental health and nutritional status of infants aged under 6 months: a secondary analysis of a cross-sectional survey
**Short Title:**	Maternal mental health and infant nutrition
**Corresponding Author:**	Mubarek Abera, PHD<br>Jimma University<br>Jimma, ETHIOPIA
**Order of Authors:**	Mubarek Abera, PHD
	Melkamu Berhane Arefayne, MD
	Carlos Grijalva-Eternod4
	Alemseged Abdissa
	Nahom Abate
	Endashaw Hailu
	Hatty Barthorp
	Elizabeth Allen
	Marie McGrath
	Tsinuel Girma
	Jonathan CK Wells
	Marko Kerac
	Emma Beaumont
**Keywords:**	maternal mental health; infant anthropometry; nutrition; growth
**Abstract:**<br><br>Need to define u6m<br>Is this supposed to be a list? In which case a : is required not ;<br>However LAZ is not anthropometry, rather derived from anthropometry (sex- and age- normalised Z-scores)<br>Or write “infant anthropometry and associated Z-Scores:” then the list<br>Rather 29.5%<br>cm<br>mm	Maternal/caregivers’ mental health (MMH) and child nutrition are both poor in low- and middle-income countries. Links between the two are plausible but poorly researched. Our aim was to inform future malnutrition management programmes by better understanding associations between MMH and the nutritional status of infants aged u6m. We conducted a health facility-based cross-sectional survey of 1060 infants in rural Ethiopia, between October 2020 and January 2021. We collected data on: MMH status (exposure) measured using the Patient Health Questionnaire (PHQ-9) and infant anthropometry (outcome); length for age Z-score (LAZ), weight for age Z-score (WAZ), weight for length Z-score (WLZ), mid upper arm circumference (MUAC), head circumference for age Z-score (HCAZ) and lower leg length (LLL). Linear regression analysis was used to determine associations between exposure and outcome variables. Mean (SD) age was 13.4 (6.2) weeks. The median score for MMH problem was 0 (inter quartile range 0 - 2) and 29.5 and 11.2% reported minimal and mild to severe depression score of 1-4 and 5-25, respectively. Mean (SD) LAZ was -0.4 (1.4), WAZ -0.7 (1.3), WLZ -0.5 (1.2), MUAC 12.4 (1.3) centimetre, HCAZ 0.4 (1.3) and LLL 148 (13.9) millimetre. In adjusted analysis, minimal MMH problems was associated with infant LAZ marginally (β=-0.2; 95% CI: -0.4, 0.001) and LLL (β=-2.0; 95% CI: -3.8, -0.1), but not with other anthropometric measurements. Significant associations were not found between mild to severe depressive symptoms and infant anthropometric outcomes. Covariates positively associated with infant anthropometric measurements were higher wealth index with LAZ (β=0.08, 95% CI: 0.03, 0.13), WAZ (β=0.12, 95% CI: 0.08, 0.17), WLZ (β=0.09, 95% CI: 0.05, 0.13), MUAC (β=0.06, 95% CI: 0.02, 0.11), and HCAZ (β=0.07, 95% CI: 0.03, 0.12); higher maternal schooling with LAZ (β=0.24, 95% CI: 0.05, 0.43) and WAZ (β=0.24, 95% CI: 0.07, 0.41); female sex with WAZ

Powered by Editorial Manager® and ProduXion Manager® from Aries Systems Corporation

	(β=0.16, 95% CI: 0.01, 0.31) and HCAZ (β=0.16, 95% CI: 0.001, 0.31); higher maternal age with LLL (β= 0.29, 95% CI: 0.07, 0.52); and improved water, sanitation and hygiene status with MUAC (β=0.07, 95% CI: 0.01, 0.12) and LLL (β=0.64, 95% CI: 0.04, 1.24). Covariates negatively associated with infant anthropometric measurements include female sex with MUAC (β=-0.33, 95% CI: -0.48, -0.18) and LLL (β=-2.51, 95% CI: -4.15, -0.87); higher household family size with WLZ (β=-0.08, 95% CI: -0.13, -0.02); exclusive breastfeeding with MUAC (β=-0.39, 95% CI: -0.55, -0.24) and LLL (β=-7.37, 95% CI: -9.01, -5.75); and grandmother family support with WAZ (β=-0.2, 95% CI: -0.3, -0.0001) and WLZ (β=-0.2, 95% CI: -0.4, 0.1). In conclusion, only minimal, but not mild, moderate or severe, maternal/caregivers' depressive symptoms are associated with infant anthropometry outcomes. Whilst plausible relationship between maternal mental health problems and offspring nutritional status exist, we are not able to show this because of small number of participants with moderate to severe level of depression in our study population. Thus, further evidence to understand and establish robust relationship between maternal mental health and offspring nutritional status is required.
**Additional Information:**	
**Question**	**Response**
**Financial Disclosure**<br><br>Enter a financial disclosure statement that describes the sources of funding for the work included in this submission and the role the funder(s) played. This includes grants and any commercial funding of the work or authors.<br><br>This statement will be typeset if the manuscript is accepted for publication.<br><br>*Please review the submission guidelines and the instructions link below for detailed requirements and guidance.*	This work is funded by Eleanor Crook Foundation (ECF)
**Competing Interests**<br><br>On behalf of all authors, disclose any competing interests that could be perceived to bias this work.<br><br>This statement will be typeset if the manuscript is accepted for publication.<br><br>*Please review the instructions link below and PLOS Global Public Health's competing interests policy to determine what information must be disclosed at*	No competing interest decaled

Powered by Editorial Manager® and ProduXion Manager® from Aries Systems Corporation

submission.	
**Data Availability** within<br><br>Before publication, Authors are required to make fully available and without restriction all data underlying their findings. Please see our PLOS Data Policy page for detailed information on this policy.<br><br>A **Data Availability Statement**, detailing where the data can be accessed, is required at first submission. Insert your Data Availability Statement in the box below.<br><br>Please see the data reporting section of our submission guidelines for instructions on what you need to include in your Data Availability Statement.<br><br>This statement will be typeset if the manuscript is accepted for publication.<br><br>*PLOS allows rare exemptions to address legal and ethical concerns. If you have legal or ethical restrictions, please use the box below to detail these in full sentences for the Journal team to consider.*	All data are available with the manuscript

Manuscript

Click here to access/download;Manuscript;MMH Infant Nutrition 2532024.V9.2.docx

Maternal mental health and nutritional status of infants aged under 6 months: a secondary analysis of a cross-sectional survey

Mubarek Abera Mengistie*[1,2], Melkamu Berhane,[2,3], Carlos S. Grijalva-Eternod[4,5], Alemseged Abdissa[6,2], Nahom Abate[7], Endashaw Hailu[7], Hatty Barthorp[8], Elizabeth Allen[9], Marie McGrath[10], Tsinuel Girma, [2,11], Jonathan CK Wells[12], Marko Kerac[4], Emma Beaumont[13]

Authors' affiliation

1Department of Psychiatry, Faculty of Medical Science, Institute of Health, Jimma University, Jimma, Ethiopia

2Jimma University Clinical and Nutrition Research Center (JUCAN)

3Department of Pediatrics and Child health, Faculty of Medical Science, Institute of Health, Jimma University, Jimma, Ethiopia

4Department of Population Health, London School of Hygiene and Tropical Medicine (LSHTM), Keppel Street, London WC1E 7HT, UK

5UCL Institute for Global Health, 30 Guilford Street, London WC1N 1EH, UK.

6Armauer Hansen Research Institute (AHRI), Addis Ababa P.O. Box 1005, Ethiopia

7GOAL Ethiopia, Bole Sub city, Woreda 06, Gurd shola, Addis Ababa, P.O. Box 5504, Ethiopia

8GOAL Global, Carnegie House, Library Road, Dun Laoghaire, A96 C7W7 Dublin, Ireland

9 Department of Medical Statistics, Faculty of Epidemiology and Population Health, London School of Hygiene and Tropical Medicine (LSHTM), Keppel Street, London WC1E 7HT, UK

10Emergency Nutrition Network, 69 High Street, Marlborough House, Kidlington, Oxfordshire OX5 2DN, UK

11Harvard Chan School of Public Health, Addis Ababa P.O. Box 1242/5646, Ethiopia

12Population, Policy and Practice Research and Teaching Department, UCL Great Ormond Street Institute of Child Health, London, United Kingdom

13Department of Infectious Disease Epidemiology and International Health, London School of Hygiene and Tropical Medicine (LSHTM), Keppel Street, London WC1E 7HT, UK

***** Correspondence: mubarek.abera@ju.edu.et, or abmubarek@gmail.com

Funding: ECF (Eleanor Crook Foundation)

ABSTRACT

See earlier comments

Maternal/caregivers' mental health (MMH) and child nutrition are both poor in low- and middle-income countries. Links between the two are plausible but poorly researched. Our aim was to inform future malnutrition management programmes by better understanding associations between MMH and the nutritional status of infants aged u6m. We conducted a health facility-based cross-sectional survey of 1060 infants in rural Ethiopia, between October 2020 and January 2021. We collected data on: MMH status (exposure) measured using the Patient Health Questionnaire (PHQ-9) and infant anthropometry (outcome); length for age Z-score (LAZ), weight for age Z-score (WAZ), weight for length Z-score (WLZ), mid upper arm circumference (MUAC), head circumference for age Z-score (HCAZ) and lower leg length (LLL). Linear regression analysis was used to determine associations between exposure and outcome variables. Mean (SD) age was 13.4 (6.2) weeks. The median score for MMH problem was 0 (inter quartile range 0 - 2) and 29.5 and 11.2% reported minimal and mild to severe depression score of 1-4 and 5-25, respectively. Mean (SD) LAZ was -0.4 (1.4), WAZ -0.7 (1.3), WLZ -0.5 (1.2), MUAC 12.4 (1.3) centimetre, HCAZ 0.4 (1.3) and LLL 148 (13.9) millimetre. In adjusted analysis, minimal MMH problems was associated with infant LAZ marginally (β=-0.2; 95% CI: -0.4, 0.001) and LLL (β=-2.0; 95% CI: -3.8, -0.1), but not with other anthropometric measurements. Significant associations were not found between mild to severe depressive symptoms and infant anthropometric outcomes. Covariates positively associated with infant anthropometric measurements were higher wealth index with LAZ (β=0.08, 95% CI: 0.03, 0.13), WAZ (β=0.12, 95% CI: 0.08, 0.17), WLZ (β=0.09, 95% CI: 0.05, 0.13), MUAC (β=0.06, 95% CI: 0.02, 0.11), and HCAZ (β=0.07, 95% CI: 0.03, 0.12); higher maternal schooling with LAZ (β=0.24, 95% CI: 0.05, 0.43) and WAZ (β=0.24, 95% CI: 0.07, 0.41); female sex with WAZ (β=0.16, 95% CI: 0.01, 0.31) and HCAZ (β=0.16, 95% CI: 0.001, 0.31); higher maternal age with LLL (β= 0.29, 95% CI: 0.07, 0.52); and improved water, sanitation and hygiene status with MUAC (β=0.07, 95% CI: 0.01, 0.12) and LLL (β=0.64, 95% CI: 0.04, 1.24). Covariates negatively associated with infant anthropometric measurements include female sex with MUAC (β=-0.33, 95% CI: -0.48, -0.18) and LLL (β=-2.51, 95% CI: -4.15, -0.87); higher household family size with WLZ (β=-0.08, 95% CI: -0.13, -0.02); exclusive breastfeeding with MUAC (β=-0.39, 95% CI: -0.55, -0.24) and LLL (β=-7.37, 95% CI: -9.01, -5.75); and grandmother family support with WAZ (β=-0.2, 95% CI: -0.3, -0.0001) and WLZ (β=-0.2, 95% CI: -0.4, 0.1). In conclusion, only minimal,

but not mild, moderate or severe, maternal/caregivers' depressive symptoms are associated with infant anthropometry outcomes. Whilst plausible relationship between maternal mental health problems and offspring nutritional status exist, we are not able to show this because of small number of participants with moderate to severe level of depression in our study population. Thus, further evidence to understand and establish robust relationship between maternal mental health and offspring nutritional status is required.

Key words: maternal mental health, infant anthropometry, nutrition, growth

INTRODUCTION

Poor level of maternal/caregiver's mental health (MMH) and poor child growth are major public health problems in low-and middle-income countries (LMICs) (1,2). Poor MMH represents a decline in the status of mental well-being during pregnancy or within a year of childbirth. Among various MMH problems, depression and anxiety during the pre-and post-natal periods are most common and increasing, particularly in LMICs. Systematic review and meta-analysis evidence showed prevalence of 25% (3) and 33% (4) peri- and post-natal depression, respectively in LMICs. Several factors such as pregnancy and child birth-related changes, role transition during and after pregnancy, intimate partner violence, low maternal social capital and poverty contribute to the high burden of MMH problems in LMICs (1). In most LMICs health care delivery systems, MMH problems are treated within the general mental health care system making the service inaccessible. Why is it inaccessible? Perhaps explain further…..

The burden of malnutrition/poor growth, manifested as stunting, wasting, and underweight, are highest in children aged under-five years in LMICs (2). Though sometimes treated separately in prevention and treatment programmes, there are common features between these different manifestations of malnutrition, most notably a short-term high mortality risk (5) and long-term risks of poor developmental outcomes and non-communicable diseases (6,7). Stunting for example starts in utero and continues during childhood (8,9). Currently, nearly 150 million children aged under-five years are stunted, mostly in LMICs (10). Stunting prevalence is as high as 50% in some settings (11), 42% in Sub Saharan Africa and 37% in Ethiopia in 2020 (2). Wasting is another major challenge associated with high case fatality and contributing to some 800,000 child-deaths per year (12). Risk factors for child malnutrition include infections and poor social, economic and environmental factors (13).

Linkages between MMH and child nutritional status are plausible. Some evidence suggest s that poor MMH is a risk factor for poor child nutrition and growth (14,15) but such evidence, especially in infants under-6 months (u6m), is scarce and complex (16–19). Some of these studies showed a negative impact of MMH problems on infant nutritional status while other reported a lack of association or presence of complex and indirect relationships between the two. More evidence on this is needed, especially since the new WHO guidelines on malnutrition highlight the need to consider MMH as part of care for infants u6m at risk of poor growth and

development (as well as older children(20)). There is increasing interest into how to integrate MMH and malnutrition programming (21) so evidence on this area are essential. in…. is

In this study, we hypothesise that poor MMH is associated with poor infant u6m nutritional status. Our aim was to examine associations between MMH problems and infant nutritional status to add new knowledge on existing evidence in a low income setting in order to inform and guide future malnutrition prevention and intervention programmes.

METHODS AND MATERIALS

Study design

This is a secondary analysis of a previously reported community-based health facility survey in Ethiopia (22). The survey was done in order to inform planning for a randomised controlled trial (23).

carried out

Setting

The original survey included health facilities in Jimma zone (food secure) and Deder district (food insecure), Ethiopia. The total population of Jimma zone is estimated to be over 3.6 million while that of Deder district is approximately 360,980 people (24). Both sites are predominantly agrarian, and their livelihood is dependent on cash crop production such as coffee and khat followed by cattle rearing. In terms of health care coverage, Jimma zone has about 124 health centres and seven hospitals and Deder district has 8 health centres. The data was collected between 12 October 2020 and 29 January 2021.

Participants

A consecutive sampling method, over a 2-week period, was employed in each of 18 health centres to recruit a total of 1060 infants u6m. Recruitment was from delivery, immunization, and growth monitoring services and under-five clinics. We included all subjects available in the original dataset. The detailed procedures and main findings of the study on anthropometric outcomes are previously reported (22).

have been

participants or infants generally preferred over subjects, which is an impersonal noun, often derogatory in nature

Measurements

Main exposure variable: MMH was assessed using the Patient Health Questionnaire (PHQ-9). Presence of mental health symptoms over the last 14 days on PHQ-9 were scored as symptom

presenting 0 = not at all, 1= over several days, 2 = more than half the days, and 3 = nearly every day. The raw sum score of individual PHQ-9 items ranges from 0 to 27. Risk of depression is classified as 0 (none), 1-4 (minimal), 5-9 (mild), 10-14 (moderate), 15-19 (moderately severe) and 20-27 (severe) depression (25). These cut offs were used in the statistical analysis.

*

Outcome variables: infant anthropometry/nutritional status (Length for age z-score (LAZ), weight for age z-score (WAZ), weight for length z-score (WLZ), mid-upper arm circumference (MUAC), head circumference for age z-score (HCAZ) and lower leg length (LLL)). Length was measured using the UNICEF length/height board to the nearest completed 0.1 cm. Weight was measured using a digital weight scale (Seca 354) to the nearest 5g if weight < 10kg or to the nearest 10g if weight ≥ 10kg. Measurement procedures followed the WHO Child Growth Standard protocols. All anthropometric indices were measured in duplicate.

l

Why mention UNICEF? Is it not a normal stadiometer? What make e.g. Seca

LLL will vary a lot between a 1 month and 5,5 month old child. To me it doesn't make sense to include anthropometric measurements in your analysis.

Household related covariates: level of maternal/caregiver education, household family size, and numbers of dependent children aged <18 years, maternal/caregiver's age, grandmother family support, household wealth status, and water and sanitation hygiene (WASH) status.

Infant related covariates: sex, age and date of birth, birth status (singleton, twin, triplet, etc), birth order, number of siblings and breastfeeding status.

Status is usually dead / alive etc
I would say e.g. Number of live births

Data analysis

The data was analysed using Stata (StataCorp. 2021) Statistical Software: Release 17.0 College Station, Texas 77845 USA: StataCorp LLC). Percentage and frequency were reported for categorical data while mean, standard deviation, median and inter quartile range (IQR) are presented for continuous data after checking normality of the distribution. Exclusive breastfeeding was considered if the mother/caregiver reported that the infant was entirely breastfed and was not given anything apart from breastmilk in the last 24 hour.

or

s

Anthropometric measurements were calculated as the average of a pair of measurements. Nutritional status for Z-scores were computed using length, weight, age and sex of the infant based on the 2006 WHO Child Growth Standards, using the *zanthro* Stata command. LAZ<-6.0 or >6.0, WAZ <-6.0 or >5.0, WLZ<-5 or >5.0 and length <45cm were considered outlier and excluded from the analysis ~~in the same order from the dataset~~.

s

On what do you base the different outlier ranges?

To test for robust relationships between MMH and infant nutritional status, unadjusted and adjusted linear regression analysis were conducted by considering different sets of confounders (infant (age, sex, birth order, and breastfeeding status), maternal (age, educational status and presence of grandmother support) and household characteristics (family size, wealth index, and water, sanitation and hygiene status)).

Ethics

Ethical approval for the original data was granted by Jimma University Institutional Review Board (ref: IHRPGD/478//2020) and by the Research Ethics Committee at the London School of Hygiene and Tropical Medicine (ref 18022). Written informed consent was obtained from the mother/caregivers (guardians) of every infant for participation. This secondary analysis, looking at mental health in detail, is directly covered by the original protocol and ethics under the secondary objective “to assess the mental health status of mothers and caregivers of surveyed newborns and infants u6m.”

RESULTS

Participants' background

From the total of 1060 participants included in the original dataset, 1036 participants were included in the current analysis. Figure 1 shows study participant flow chart.

Figure 1 here

Of those analysed, 577 (55.3%) infants were male and 262 (25.1%) were first borns. Infant's mean age (SD) was 12.8 (±6.2) weeks. Mean (SD) maternal/caregiver's age was 25.9 (±5.4) years, and more than half of the mothers/caregivers 619 (59.4%) had attended at least some level of formal education. Nearly 60% of the households had family size of five or less.

Table 1 here

Exposure and outcome characteristics

In terms of MMH, 920 (88.8%) of the mother/caregiver scored none-minimal depression [614 (59.3% no depression and 306 (29.5%) minimal depression], while the rest 116 (11.2%) scored mild to severe depression [78 (7.5%) mild, 23 (2.2%) moderate, 8 (0.8%) moderately severe, and 7 (0.7%) severe depression]. Figure 2 show MMH histogram. The median MMH score was 0 and IQR of 0-2.

Figure 2 here

The overall mean and 95% confidence interval (CI) for infant nutritional status was LAZ -0.4 (-0.4, -0.3), WAZ -0.7 (-0.7, -0.6) and WLZ -0.5 (-0.6, -0.4). All were below the mean value for WHO reference data. The mean (95% CI) value for MUAC was 12.4cm (12.3, 12.5), HCAZ 0.4 (0.3, 0.5) and LLL 148mm (147.1, 148.9). Comparison for infants' nutritional status by level of maternal mental health is presented in table 1. Figure 3 shows scatter plot between MMH problem score and infant LAZ.

Re-phrase. This sounds like only the problem scores are shown, whereas all scores are shown in Figure 3. Name the tool rather.

Figure 3 here

Association between MMH and infant nutritional status

Most are Z-scores so you need to add e.g. "and associated Z-scores"

In Table 2 we reported associations of MMH and infant anthropometric measurements. In unadjusted statistical analysis, only minimal depression (PHQ score 1-4) compared to no depression (PHQ score 0) was negatively associated with LAZ (β=-0.2; 95% CI: -0.4, -0.01) and LLL (β=-2.0, 95% CI: -3.9, -0.1), but not with other anthropometric measurements: WAZ (β=-0.1; 95% CI: -0.3, 0.04), WLZ (β=0.02; 95% CI: -0.2, 0.2), MUAC (β=-0.1, 95% CI: -0.3, 0.1) and head circumference (β=-0.1, 95% CI: -0.3, 0.1). In the final adjusted statistical analysis, minimal maternal depression was associated negatively with LAZ (β=-0.2; 95% CI: -0.4, 0.0) marginally and LLL (β=-2.0; 95% CI: -3.9, -0.1) and PHQ score 1-27 was associated with LLL ((β=-1.8; 95% CI: -3.4, -0.1). In both unadjusted and adjusted statistical analysis, significant associations were not found between mild, moderate, moderately severe, and severe level of depression with any of infant anthropometric measurements (LAZ, WAZ, WLZ, MUAC, HCAZ and LLL).

Are these associations significant though? You need to add P-values.

Covariates positively associated with infant anthropometric measurements were higher wealth index with LAZ (β=0.08, 95% CI: 0.03, 0.13), WAZ (β=0.12, 95% CI: 0.08, 0.17), WLZ (β=0.09, 95% CI: 0.05, 0.13), MUAC (β=0.06, 95% CI: 0.02, 0.11), and HCAZ (β=0.07, 95% CI: 0.03, 0.12); higher maternal schooling with LAZ (β=0.24, 95% CI: 0.05, 0.43) and WAZ (β=0.24, 95% CI: 0.07, 0.41); female sex with WAZ (β=0.16, 95% CI: 0.01, 0.31) and HCAZ (β=0.16, 95% CI: 0.001, 0.31); higher maternal age with LLL (β= 0.29, 95% CI: 0.07, 0.52); and improved water, sanitation and hygiene status with MUAC (β=0.07, 95% CI: 0.01, 0.12) and LLL (β=0.64, 95% CI: 0.04, 1.24).

Are these associations significant though? You need to add P-values.

Covariates negatively associated with infant anthropometric measurements include female sex with MUAC (β=-0.33, 95% CI: -0.48, -0.18) and LLL (β=-2.51, 95% CI: -4.15, -0.87); higher household family size with WLZ (β=-0.08, 95% CI: -0.13, -0.02); exclusive breastfeeding with MUAC (β=-0.39, 95% CI: -0.55, -0.24) and LLL (β=-7.37, 95% CI: -9.01, -5.75); and grandmother family support with WAZ (β=-0.2, 95% CI: -0.3, -0.0001) and WLZ (β=-0.2, 95% CI: -0.4, 0.1).

Are these associations significant though? You need to add P-values.

Table 2 here

Discussion

In this study we examined associations between maternal/caregiver's mental health with infant nutritional status as indicated by anthropometry. The prevalence of maternal depressive symptoms (PHQ score 5-27) is only 11.2% and the mean (SD) nutritional status of infants u6m was LAZ -0.4 (1.4), WAZ -0.7 (1.3), WLZ -0.5 (1.2), MUAC 12.4 cm (1.3), HCAZ 0.4 (1.3) and LLL is 148 mm (14.0). The prevalence of maternal depressive symptom is lower than previous reports including from other studies in Ethiopia (26,27). Mild, moderate, moderately severe and severe depressive symptoms showed no association with any of the nutritional status indicators. Only minimal depressive symptom associated with low LAZ marginally and low LLL but not with other anthropometric measurements.

The lack of associations between higher maternal depressive symptoms with infant nutritional status is consistent with previous studies where maternal common mental problems were not associated with nutritional status of under five years children in Ethiopia (14,28,29), but contrasts with several other studies from LMICs (16–19,30–35) including findings from Ethiopia (36,37). Our finding is consistent with findings of high income settings (European cohort study (38) and the generation R study (39)) and upper middle income countries (e.g. Brazil) (40) where higher maternal depressive symptoms showed no association with children nutritional status (38–40). In general, associations between postnatal MMH problems and poor child nutritional status have been mainly found in LMICs while lack of associations are mainly from high income setting (15). Unlike our study, previous studies did not included tests of associations between minimal depressive symptoms and childhood nutritional status. Furthermore, the intensity of relationship and direction of associations between these two major public health problems are not well established.

Previously reported associations between maternal mental health problems and poor childhood nutritional status (16–19,30–35) can be explained by several causal mechanisms involving poor maternal child care practice and episodes of childhood illness (4,15,41). Maternal depressive symptoms associated with poor infant feeding practices (42), poor infant breastfeeding outcomes including shorter duration of exclusive breastfeeding (15), poor mother-to-infant bonding (15), low oxytocin total T4 concentration (43) and low breastmilk secretory immunoglobulin A

Perhaps some context here on Ethiopia, are most babies breastfed etc.

concentration (44) in the breastmilk. Children especially in their early life are entirely dependent on their mother/caregiver's competencies and resources for nutrition, survival, growth and development. Maternal postnatal depression affects maternal ability and competences to provide responsive and supportive care to her baby (15). This could result in poor maternal-child attachment, bonding, breastfeeding and stimulation; all of which could affect offspring nutrition and growth. Moreover, depressed mothers have poor emotional sensitivity and responsiveness to their environment including their babies (15) which may put at risk infant without support. Nurturing care as proposed by UNICEF and WHO for child growth and development requires 5 domains of care (good health, adequate nutrition, safety and security, responsive care and early opportunity for learning) (45). All of these domains require good maternal mental health and competencies to manage holistic aspects of her child's condition.

Re-phrase

However these causal pathways through which MMH problems impact offspring nutritional status and our proposed hypothesis are not supported in the current findings. Moreover the lack of association between MMH problems and poor child nutritional status in the previous three studies from Ethiopia (14,28,29) and from upper middle income and high income countries were (38–40) not adequately discussed. Several factors such as the setting and context, how the data was collected, types of screening tool used, and the skills, competencies and confidence of data collectors in asking sensitive questions in mental health could have all played a role for the lack of association between MMH problems and poor child nutritional status.

I think reported rather than discussed

Implication of the finings

Despite plausible relation between MMH problems and poor infant/child nutritional status exist, our data do not support this. The lack of association in the current study by itself is not evidence of an absent link between the two problems. Moreover, the lack of association between severe MMH problems and infants nutritional status in the current study may be explained by the low prevalence of moderate to severe depression; though previous studies from Ethiopia had failed to establish an association despite the high prevalence of maternal depressive symptoms ~~were~~ reported. Depressive disorder by its nature is unique in its clinical presentation compared to many other psychiatric illnesses in that most patients with depression have a good level of insight and understanding about their ill mental health status. This may lead to under or over reporting based on the context and circumstances how and where the screening process takes place. The high

Re-phrase, this is not English

Perhaps "poor" is better here

prevalence of minimal depressive symptoms over the low prevalence of moderate to severe depression may indicate that majority of severely affected participants could minimize symptoms due to lack of confidence to report symptoms in a non-conducive screening environment. Screening for depression requires an established rapport and therapeutic relationship which could take longer time or repeated visits to encourage clients to report their problem by resisting stigma and discrimination. These could have implication to future integration of MMH care in to the maternal and child health service which are too crowded to ensure adequate assessment, privacy and confidentiality. While there is increasing interest into how to integrate MMH and malnutrition programming (21), screening strategy for MMH in the management of small and nutritionally at-risk infants aged under 6 months and their mothers (MAMI) context may require a unique rather than the routine mental health screening approach to be able to effectively elicit symptoms. In general additional qualitative, epidemiological and experimental studies are essential to understand various circumstances that could explain the current finings to reach on robust conclusion.

Strengths and limitations

We have focused on young infants (u6m) where there is a lack of evidence on this area to support clear decision making process. We covered two different geographic settings representing food secure and insecure areas. Moreover we have included large number of study participants. This study however is not without limitations. The study is a facility based survey and therefore may not be representative of the general population. Moreover the setting where MMH data were collected within the maternal and child health unit may not be conducive enough to establish trusted relationship between participants and study nurses. Finally, though PQH-9 has been widely used in LMIC settings, including in Ethiopia, it may not be optimal in identifying MMH symptoms in the MAMI context and other screening tools/strategies may be more valid and may have shown different association with anthropometric deficit.

CONCLUSIONS

Mild to moderate and severe levels of maternal/caregiver's mental health problems are not associated with poor infant nutritional status. Whilst there is plausible relationship between MMH problems and offspring nutritional status, this hypothesis is not supported in the current study. Thus, further evidence to understand and establish the relationship between postnatal

maternal mental health problems and infant u6m nutritional status is required both in Ethiopia and other LMIC settings.

REFERENCES

1. McNab SE, Dryer SL, Fitzgerald L, Gomez P, Bhatti AM, Kenyi E, et al. The silent burden: a landscape analysis of common perinatal mental disorders in low- and middle-income countries. BMC Pregnancy Childbirth. 2022 Dec;22(1):342.

2. Local Burden of Disease Child Growth Failure Collaborators. Mapping child growth failure across low- and middle-income countries. Nature. 2020 Jan 9;577(7789):231–4.

3. Roddy Mitchell A, Gordon H, Lindquist A, Walker SP, Homer CSE, Middleton A, et al. Prevalence of Perinatal Depression in Low- and Middle-Income Countries: A Systematic Review and Meta-analysis. JAMA Psychiatry [Internet]. 2023 Mar 8 [cited 2023 Mar 28]; Available from: https://jamanetwork.com/journals/jamapsychiatry/fullarticle/2802140

4. Dadi AF, Miller ER, Mwanri L. Postnatal depression and its association with adverse infant health outcomes in low- and middle-income countries: a systematic review and meta-analysis. BMC Pregnancy Childbirth. 2020 Dec;20(1):416.

5. Kerac M, McGrath M, Connell N, Kompala C, Moore WH, Bailey J, et al. ‘Severe malnutrition’: thinking deeplyS, communicating simply. BMJ Glob Health. 2020 Nov;5(11):e003023.

6. Kirolos A, Goyheneix M, Kalmus Eliasz M, Chisala M, Lissauer S, Gladstone M, et al. Neurodevelopmental, cognitive, behavioural and mental health impairments following childhood malnutrition: a systematic review. BMJ Glob Health. 2022 Jul;7(7):e009330.

7. Grey K, Gonzales GB, Abera M, Lelijveld N, Thompson D, Berhane M, et al. Severe malnutrition or famine exposure in childhood and cardiometabolic non-communicable disease later in life: a systematic review. BMJ Glob Health. 2021 Mar;6(3):e003161.

8. Gonete AT, Kassahun B, Mekonnen EG, Takele WW. Stunting at birth and associated factors among newborns delivered at the University of Gondar Comprehensive Specialized Referral Hospital. Petry CJ, editor. PLOS ONE. 2021 Jan 20;16(1):e0245528.

9. Fantay Gebru K, Mekonnen Haileselassie W, Haftom Temesgen A, Oumer Seid A, Afework Mulugeta B. Determinants of stunting among under-five children in Ethiopia: a multilevel mixed-effects analysis of 2016 Ethiopian demographic and health survey data. BMC Pediatr. 2019 Dec;19(1):176.

10. United Nations Children’s Fund (UNICEF), World Health Organization, International Bank for Reconstruction and Development/The World Bank. Levels and trends in child malnutrition: key findings of the 2021 edition of the joint child malnutrition estimates. Geneva: World Health Organization; 2021. Licence: CC BY-NC-SA 3.0 IGO.

11. Aboagye RG, Ahinkorah BO, Seidu AA, Frimpong JB, Archer AG, Adu C, et al. Birth weight and nutritional status of children under five in sub-Saharan Africa. Kumar S, editor. PLOS ONE. 2022 Jun 9;17(6):e0269279.

12. Black RE, Morris SS, Bryce J. Where and why are 10 million children dying every year? The Lancet. 2003 Jun;361(9376):2226–34.

13. Amir-ud-Din R, Fawad S, Naz L, Zafar S, Kumar R, Pongpanich S. Nutritional inequalities among under-five children: a geospatial analysis of hotspots and cold spots in 73 low- and middle-income countries. Int J Equity Health. 2022 Sep 15;21(1):135.

14. Harpham T. Maternal mental health and child nutritional status in four developing countries. J Epidemiol Community Health. 2005 Dec 1;59(12):1060–4.

15. Slomian J, Honvo G, Emonts P, Reginster JY, Bruyère O. Consequences of maternal postpartum depression: A systematic review of maternal and infant outcomes. Womens Health. 2019 Jan;15:174550651984404.

16. Stewart RC. Maternal depression and infant growth ? a review of recent evidence. Matern Child Nutr. 2007 Apr;3(2):94–107.

17. Black MM, Baqui AH, Zaman K, Arifeen SE, Black RE. Maternal depressive symptoms and infant growth in rural Bangladesh. Am J Clin Nutr. 2009 Mar;89(3):951S-957S.

18. Patel V. Postnatal depression and infant growth and development in low income countries: a cohort study from Goa, India. Arch Dis Child. 2003 Jan 1;88(1):34–7.

19. Khan AM. Maternal mental health and child nutritional status in an urban slum in Bangladesh: A cross-sectional study. Amugsi DA, editor. PLOS Glob Public Health. 2022 Oct 19;2(10):e0000871.

20. WHO guideline on the prevention and management of wasting and nutritional oedema (acute malnutrition) in infants and children under 5 years.

21. Mee N, Abera M, Kerac M. Acceptability and Feasibility of Maternal Mental Health Assessment When Managing Small, Nutritionally At-Risk Infants Aged < 6 Months: A Key Informant Interview Study. Children. 2024 Feb 6;11(2):209.

22. Grijalva-Eternod CS, Beaumont E, Rana R, Abate N, Barthorp H, McGrath M, et al. Malnutrition in Infants Aged under 6 Months Attending Community Health Centres: A Cross Sectional Survey. Nutrients. 2021 Jul 21;13(8):2489.

23. Kerac M. Testing a new clinical care pathway for the management of small and nutritionally at-risk infants aged under 6 months and their mothers [Internet]. [cited 2024 Mar 21]. Available from: https://www.isrctn.com/ISRCTN47300347

24. Ethiopian statistical service: https://www.statsethiopia.gov.et/wp-content/uploads/2023/08/Population-of-Zones-and-Weredas-Projected-as-of-July-2023.

25. Kroenke K, Spitzer RL, Williams JBW. The PHQ-9: Validity of a brief depression severity measure. J Gen Intern Med. 2001 Sep;16(9):606–13.

26. Hanlon C. Maternal depression in low- and middle-income countries. Int Health. 2013 Mar 1;5(1):4–5.

27. Tolossa T, Fetensa G, Yilma MT, Abadiga M, Wakuma B, Besho M, et al. Postpartum depression and associated factors among postpartum women in Ethiopia: a systematic review and meta-analysis, 2020. Public Health Rev. 2020 Dec;41(1):21.

28. Nguyen PH, Saha KK, Ali D, Menon P, Manohar S, Mai LT, et al. Maternal mental health is associated with child undernutrition and illness in Bangladesh, Vietnam and Ethiopia. Public Health Nutr. 2014 Jun;17(6):1318–27.

29. Dadi AF, Miller ER, Woodman RJ, Azale T, Mwanri L. Effect of perinatal depression on risk of adverse infant health outcomes in mother-infant dyads in Gondar town: a causal analysis. BMC Pregnancy Childbirth. 2021 Dec;21(1):255.

30. Wemakor A, Mensah KA. Association between maternal depression and child stunting in Northern Ghana: a cross-sectional study. BMC Public Health. 2016 Dec;16(1):869.

31. Surkan PJ, Kennedy CE, Hurley KM, Black MM. Maternal depression and early childhood growth in developing countries: systematic review and meta-analysis. Bull World Health Organ. 2011 Aug 1;89(8):608-615E.

32. Ndokera R, MacArthur C. The relationship between maternal depression and adverse infant health outcomes in Zambia: a cross-sectional feasibility study: Maternal depression and adverse infant health outcomes in Zambia. Child Care Health Dev. 2011 Jan;37(1):74–81.

33. Avan B, Richter LM, Ramchandani PG, Norris SA, Stein A. Maternal postnatal depression and children's growth and behaviour during the early years of life: exploring the interaction between physical and mental health. Arch Dis Child. 2010 Sep 1;95(9):690–5.

34. Bakare MO, Okoye JO, Obindo JT. Introducing depression and developmental screenings into the National Programme on Immunization (NPI) in southeast Nigeria: an experimental cross-sectional assessment. Gen Hosp Psychiatry. 2014 Jan;36(1):105–12.

35. Ashaba S, Rukundo GZ, Beinempaka F, Ntaro M, LeBlanc JC. Maternal depression and malnutrition in children in southwest Uganda: a case control study. BMC Public Health. 2015 Dec;15(1):1303.

36. Anato A, Baye K, Tafese Z, Stoecker BJ. Maternal depression is associated with child undernutrition: A cross- sectional study in Ethiopia. Matern Child Nutr. 2020 Jul;16(3):e12934.

37. Girma S, Fikadu T, Abdisa E. Maternal Common Mental Disorder as Predictors of Stunting among Children Aged 6-59 Months in Western Ethiopia: A Case-Control Study. Int J Pediatr. 2019 Mar 10;2019:1–8.

38. Grote V, Vik T, von Kries R, Luque V, Socha J, Verduci E, et al. Maternal postnatal depression and child growth: a European cohort study. BMC Pediatr. 2010 Dec;10(1):14.

39. Ertel KA, Kleinman K, Van Rossem L, Sagiv S, Tiemeier H, Hofman A, et al. Maternal perinatal depression is not independently associated with child body mass index in the Generation R Study: methods and missing data matter. J Clin Epidemiol. 2012 Dec;65(12):1300–9.

40. Lima TFM, Maciel WM, Alencar MND, Cruz JADS, Carvalho CAD, Silva AAMD. Association between maternal depressive symptoms with child malnutrition or child excess weight. Rev Bras Saúde Materno Infant. 2017 Jul;17(3):591–601.

41. Dadi AF, Akalu TY, Wolde HF, Baraki AG. Effect of perinatal depression on birth and infant health outcomes: a systematic review and meta-analysis of observational studies from Africa. Arch Public Health. 2022 Dec;80(1):34.

42. Woldetensay YK, Belachew T, Ghosh S, Kantelhardt EJ, Biesalski HK, Scherbaum V. The effect of maternal depressive symptoms on infant feeding practices in rural Ethiopia: community based birth cohort study. Int Breastfeed J. 2021 Dec;16(1):27.

43. Stuebe AM, Grewen K, Meltzer-Brody S. Association Between Maternal Mood and Oxytocin Response to Breastfeeding. J Womens Health. 2013 Apr;22(4):352–61.

44. Kawano A, Emori Y. The Relationship Between Maternal Postpartum Psychological State and Breast Milk Secretory Immunoglobulin A Level. J Am Psychiatr Nurses Assoc. 2015 Jan;21(1):23–30.

45. World Health Organization. Nurturing care for early childhood development: a framework for helping children survive and thrive to transform health and human potential [Internet]. Geneva: World Health Organization; 2018 [cited 2023 Mar 29]. 55 p. Available from: https://apps.who.int/iris/handle/10665/272603

Tables

Table 1: Background characteristics of study participants, n=1036			
**Characteristics**	**Category**	**n(%), mean ± SD**	
Infant sex	Male	574 (55.4)	
Infant age	Weeks	13.4 ±6.2	
Infant birth order	1st born	259 (25.0)	
	2nd born	214 (20.7)	
	3rd born	156 (15.1)	
	4th born	127 (12.3)	
	5th born	116 (11.2)	
	>5th born	164 (15.8)	
Primary caregiver's age, years		25.9 ±5.4	
Primary caregiver schooling	Ever attended school	612 (59.1)	
Household size	1-4	441 (42.6)	
	5-6	306 (29.5)	
	>6	289 (27.9)	
Maternal/caregivers mental health	PHQ score 0	614 (59.3)	
	PHQ score 1-4	306 (29.5)	
	PHQ score 5-9	78 (7.5)	
	PHQ score 10-14	23(2.2)	
	PHQ score 15-19	8 (0.8)	
	PHQ score 20-27	7 (0.7)	
Infant nutritional status	All participants (N=1036)	Poor maternal mental health (PHQ 5-27) (n=116)	Maternal mental health (PHQ 0-5) (n=920)
Length for age Z-score	-0.35 ±1.4	-.25± 1.25	-.35± 1.37
Weight for age Z-score	-0.65 ±1.3	-.65± 1.27	-.65± 1.27
Weight for length for age Z-score	-0.48 ±1.2	-.59± 1.22	-.47± 1.24
MUAC, centimetre	12.4± 1.3	12.31± 1.34	12.45± 1.25
Head circumference Z-score	0.41± 1.3	.52 ± 1.42	.40± 1.25
Lower leg length, millimetre	148.07± 13.92	149.07± 16.54	147.06± 13.6

Ref not Reff

Table 2: Associations between MMH and infant nutritional status in unadjusted and adjusted statistical models, n=1036

**Unadjusted model**	**LAZ**			**WAZ**			**WLZ**			**MUAC**			**Head circumference**			**Lower leg length**		
	β	95% CI		β	95% CI		β	95% CI		β	95% CI		β	95% CI		β	95% CI	
Reff: PHQ score =0										0			0			0		
PHQ 1-4	**-0.20**	**-0.38**	**-0.01**	-0.14	-0.31	0.04	0.02	-0.15	0.19	-0.08	-0.25	0.10	-0.13	-0.31	0.05	**-1.95**	**-3.86**	**-0.04**
PHQ 5-9	-0.05	-0.36	0.27	-0.03	-0.33	0.27	0.005	-0.29	0.30	-0.15	-0.44	0.15	0.08	-0.22	0.38	-1.92	-5.20	1.35
PHQ 10-14	0.35	-0.22	0.91	-0.05	-0.58	0.48	-0.49	-1.00	0.03	-0.15	-0.68	0.38	0.11	-0.42	0.64	2.12	-3.68	7.91
PHQ 15-19	0.09	-0.87	1.03	0.30	-0.59	1.18	0.28	-0.59	1.14	-0.23	-1.11	0.66	-0.02	-0.91	0.87	1.91	-7.80	11.63
PHQ 20-27	-0.17	-1.19	0.84	-0.27	-1.22	0.67	-0.14	-1.07	0.78	-0.38	-1.32	0.57	0.01	-0.94	0.96	3.07	-7.30	13.45
PHQ-1-27	-0.13	-0.30	0.04	-0.11	-0.26	0.05	-0.01	-0.16	0.14	-0.10	-0.26	0.05	-0.07	-0.23	0.08	**-1.56**	**-3.29**	**-0.16**
Reff: PHQ score 0-4	0			0			0			0			0			0		
PHQ score 5-9	0.02	-0.30	0.33	0.02	-0.28	0.31	-0.002	-0.29	0.28	-.12	-0.41	0.17	0.12	-0.17	0.42	-1.28	-4.50	1.95
PHQ score 10-14	0.41	-0.15	0 .98	-0.01	-0.53	0.52	-0.49	**-1.01**	**0.02**	-.12	-0.65	0.40	0.15	-0.38	0.68	2.76	-3.01	8.53
PHQ score 15-19	0.15	-0.80	1.10	0.35	-0.54	1.23	0.27	-0.59	1.13	-.20	-1.08	0.68	0.02	-0.86	0.91	2.56	-7.15	12.27
PHQ score 20-27	-0.11	-1.12	0.91	-0.23	-1.17	0.72	-0.15	-1.07	0.77	-.35	-1.29	0.59	0.05	-0.89	1.00	3.72	-6.65	14.09
PHQ score 5-27	0.10	-0.17	0.36	0.02	-0.22	0.27	-0.09	-0.33	0.15	-.14	-0.38	0.10	0.12	-0.13	0.36	0.09	-2.60	2.78
**Final adjusted model -a**	**LAZ**			**WAZ**			**WLZ**			**MUAC**			**Head circumference**			**Lower leg length**		
	β	[95% CI]		β	[95% CI]		β	[95% CI]		β	[95% CI]		β	[95% CI]		B	[95% CI]	
**Reff. PHQ score =0**	0			0			0			0			0			0		
PHQ score 1-4	**-0.19**	**-0.37**	**0.001**	-0.13	-0.30	0.04	0 .02	-0.15	0.19	-0.07	-0.24	0 .09	-0.12	-0.29	0 .05	**-1.95**	**-3.78**	**-0.13**
PHQ score 5-9	-0.04	-0.36	0.28	0 .03	-0.26	0 .33	0.09	-0.20	0.38	-0.11	-0.40	0.18	0.08	-0.21	0.38	-2.34	-5.48	0.81
PHQ score 10-14	0.32	-0.25	0.88	-0.01	-0.52	0.51	-0.38	-0.89	0.13	-0.21	-0.72	0.31	0.07	-0.46	0 .59	0 .57	-4.99	6.13
PHQ score 15-19	0.02	-0.93	0.96	0.28	-0.59	1.14	0.33	-0.52	1.18	-0.16	-1.02	0.70	-0.09	-0.97	0.78	2.71	-6.56	11.98
PHQ score 20-27	-0.17	-1.18	0.84	-0.14	-1.07	0.78	0.04	-0.87	0.95	-0.42	-1.33	0 .49	0.003	-0.94	0 .94	0.80	-9.10	10.71
PHQ score 1-27	-0.13	-0.29	0.04	-0.09	-0.24	0.07	0.02	-0.13	0.17	-0.09	-0.25	0 .06	-0.07	-0.23	0 .09	**-1.76**	**-3.41**	**-0.11**
**Final adjusted model -b**																		
**Reff: PHQ score 0-4**	0			0			0			0			0			0		
PHQ score 5-9	0.02	-0.29	0.33	0.07	-0.21	0 .36	0.08	-0.20	0.36	-0.08	-0.36	0 .20	0.13	-0.17	0.42	-1.68	-4.77	1.41
PHQ score 10-14	0.38	-0.18	0.94	0.04	-0.48	0.55	-0.39	-0.90	0.12	-0.18	-0.69	0.33	0.11	-0.42	0.63	1.22	-4.31	6.75
PHQ score 15-19	0.08	-0.86	1.02	0.32	-0.54	1.19	0.32	-0.53	1.17	-0.13	-1.00	0 .72	-0.05	-0.93	0 .83	3.38	-5.88	12.65
PHQ score 20-27	-0.11	-1.11	0.90	-0.10	-1.02	0.82	0.03	-0.87	0.94	-0.39	-1.31	0.52	0.04	-0.89	0.98	1.47	-8.43	11.37
PHQ score 5-27	0.09	-0.18	0.35	0.07	-0.17	0.32	0.002	-0.24	0 .24	-0.12	-0.36	0 .12	0.10	-0.14	0.35	-0.58	-3.17	2.01
Adjusted Covariates																		
Female infant	0.16	-0.01	0.33	**0.16**	**0.01**	**0.31**	0.10	-0.05	0.25	**-0.33**	**-0.48**	**-0.18**	**0.16**	**0.001**	**0.31**	**-2.51**	**-4.15**	**-0.87**
Increasing birth order	0.11	0.03	0.20	**0.10**	-0 .02	0.17	0.01	-0.06	0.09	0.05	-0.02	0.12	**0.13**	**0.05**	**0.21**	0.72	-0.10	1.54
Exclusive breastfeeding	0.11	-0.06	0.28	0.11	-0.04	0.26	0.03	-0.12	0.18	**-0.39**	**-0.56**	**-0.24**	0 .04	-0.11	0.20	**-7.37**	**-9.01**	**-5.75**
Mother's /primary caregiver's age	-0.02	-0.04	.01	-0.01	-0.03	0.01	0.01	-0.01	0.03	0.02	-0.01	0.04	-0.006	-0.03	0.02	**0.29**	**0.07**	**0.52**
primary caregiver attended schooling	**0.24**	**0.05**	**0.43**	**0.24**	**0.07**	**0.41**	0.07	-0.10	0.24	0.19	-0.01	0.36	0.12	-0.06	0.30	1.04	-0.83	2.90
Increasing household family size	-0.001	-0.07	0.07	-0.05	-0.11	0.01	**-0.08**	**-0.13**	**-0.02**	**-0.06**	**-0.12**	**-0.001**	-0.05	-0.11	0.01	-0.64	-1.29	0.01
Grand parent support	-0.01	-0.19	0.17	**-0.16**	**-0.33**	**-0.0001**	**-0.22**	**-0.38**	**-0.06**	-0.10	-0.26	0.06	-0.09	-0.26	0.08	-0.55	-2.30	1.21
Increasing wealth status	**0.08**	**0.02**	**0.13**	**0.12**	**0.08**	**0.17**	**0.09**	**0.05**	**0.14**	**0.06**	**0.02**	**0.11**	**0.07**	**0.03**	0.12	0.10	-0.58	0.39
Increasing WASH status	0.004	-0.06	0.06	0.01	-0.04	0 .07	0.01	-0.05	0.06	**0.07**	**0.01**	**0.12**	0.01	-0.05	0.06	**0.64**	**0.04**	**1.24**

I presume the bolded values are the significant ones, please state this in the footnote.

***Note**: Final adjusted model –a (comparator no depression) and final adjusted model- b (comparator no to minimal depression) are final regression model both adjusted for infant sex, birth order, exclusive breastfeeding status, mother/caregivers' age and schooling, household family size, availability of grand parent support, wealth status, and WASH status. Outputs for covariates are from final model-b.*

Figure
Click here to access/download;Figure;Figure 1.docx

Figure 1

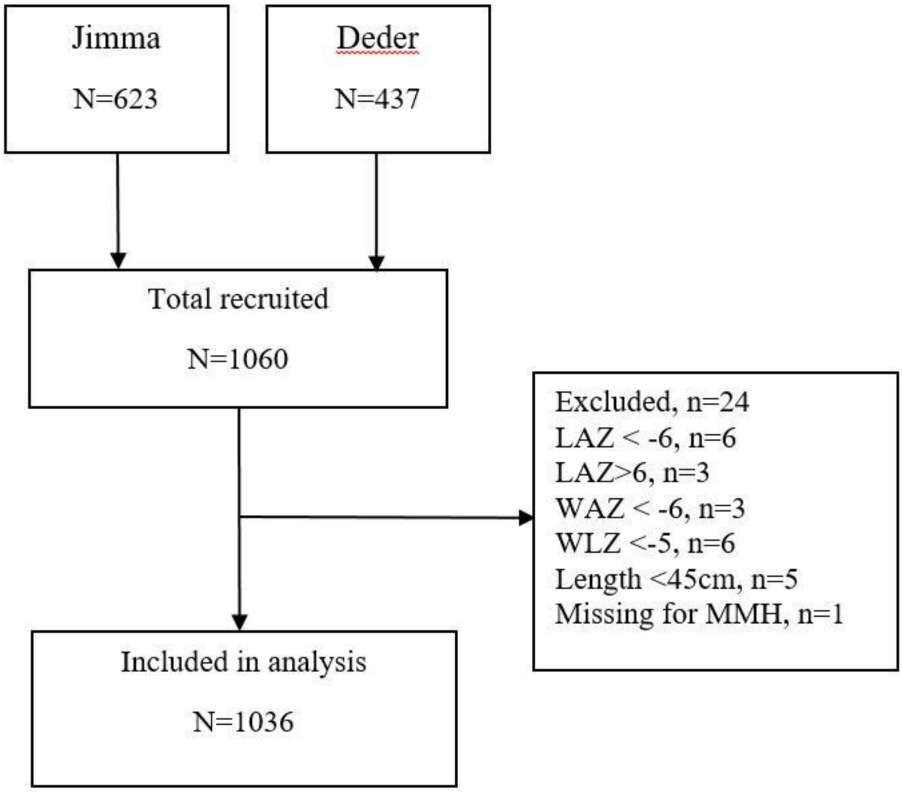

Spacing inconsistent with < and >

Figure 1: Study participant flow chart

Figure 2

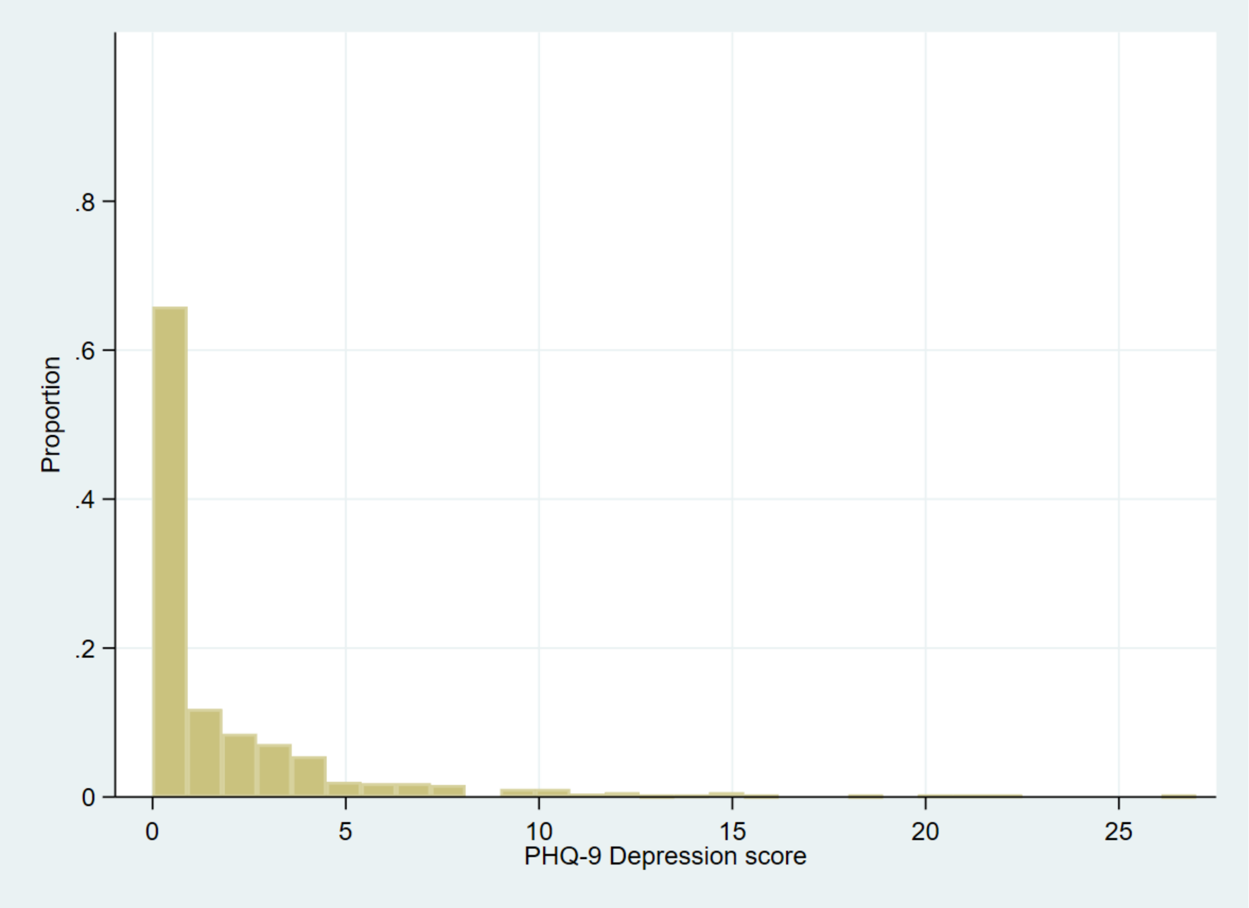

Figure 2: Maternal mental health score histogram

Click here to access/download;Figure;Figure 3.docx

Figure 3

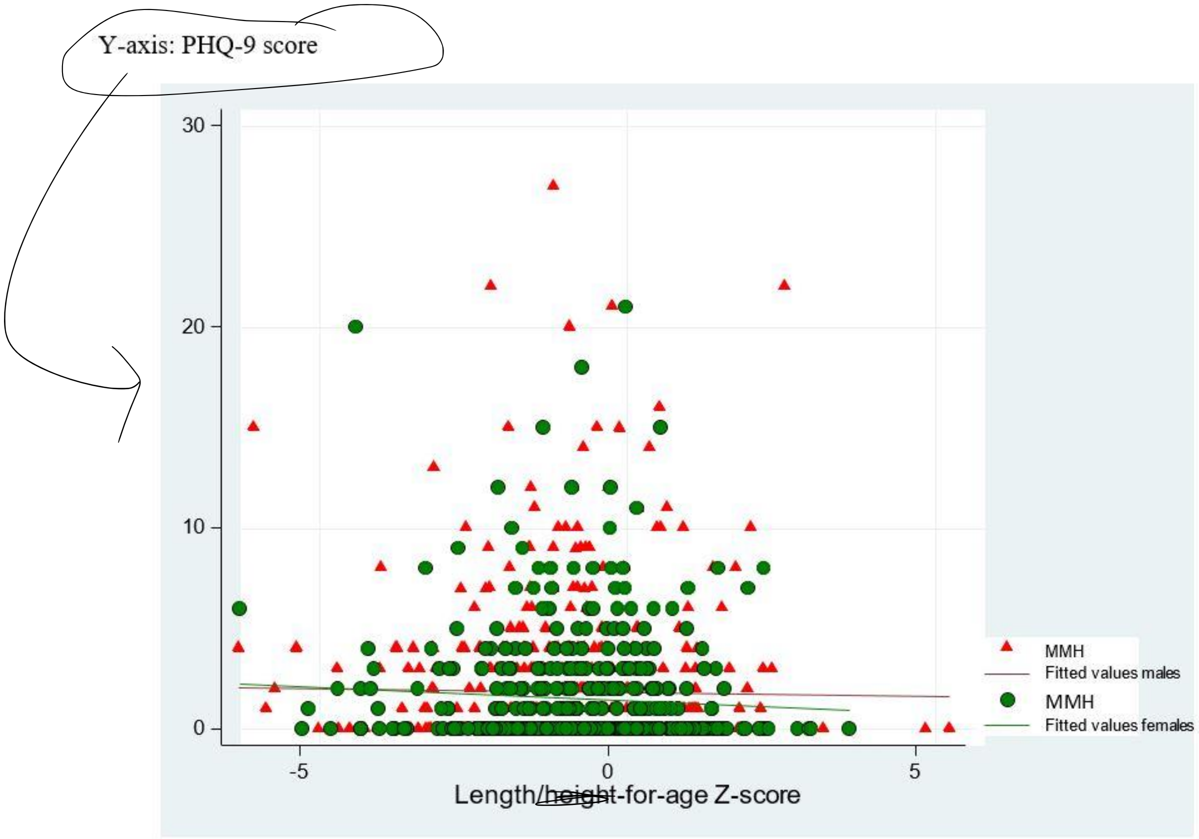

Figure 3: Scatter plot between PHQ-9 score and infant Length for age Z-score
