# Supplementary material for: Maternal mental health and nutritional status of infants aged under 6 months: A secondary analysis of a cross-sectional survey
Source: PLOS Glob Public Health. 2024 Sep 13;4(9):e0003139. doi: 10.1371/journal.pgph.0003139 (PMC11398659; doi:10.1371/journal.pgph.0003139)
Supplement: Attachment — Submitted filename: Rebutal letter.docx [file pgph.0003139.s004.docx]

**Response to reviewer’s comment**

**General response:**

The authors are thankful to the editor and reviewers for taking their time to review and give us important feedback/comments on our manuscript for improvement. We have considered the comments and have done the necessary revision and edits throughout the document. Below please find point-by-point response to the issues raised. We have used track change in the main document to indicate changes.

**Specific response**

1. **Reviewer 1.**
   1.1. Abstract: ‘u6m’ should be spelled out on first use.

**Response:** This is now corrected as;

*“under six month (u6m)”*

1.2. Introduction: The definition of poor MMH (line 2-3) should be referenced.

**Response:** Now the following reference is *added;*

*“American Psychiatric Association, DSM-5 Task Force. (2013).* Diagnostic and statistical manual of mental disorders: DSM-5™ *(5th ed.). American Psychiatric Publishing, Inc.* [*https://doi.org/10.1176/appi.books.9780890425596*](https://psycnet.apa.org/doi/10.1176/appi.books.9780890425596)*”*

1.3. Methods: A list of recruitments sites is presented. Please clarify how these sites were sampled/weighted as each is likely to represent a different group of infants. For example, neonates at delivery sites, well-children at immunization sites, malnourished children attending growth-monitoring services etc. What were the inclusion and exclusion criteria?

**Response**: All infants u6m in each of the recruitment sites visiting the health facility over the 2 weeks screening period were included in the study. All identified infants in each of the recruitment sites are collected/invited to come to one assessment unit in that specific health facility to carry out the anthropometric measurements and questionnaire based assessments. While there is no specific exclusion criteria, all infant attending health facility for one of several reasons were included in the study. For further understanding - detail of the original study is published and can be accessed at <https://www.mdpi.com/2072-6643/13/8/2489>

1.4. Comment: Wealth index is noted in the Abstract and Tables but it is not defined.

**Response**: thank you for this comment. Now we have clarified this *as*

*“Wealth index was computed by employing principal component analysis considering ownership to house, diesel water pump, radio, television, watch/clock, mobile phone, land line (non-mobile) phone, refrigerator, table, chair, sponge matrix, straw matrix, grass matrix, electrical stove, kerosene stove, kerosene lamp, press lamp, bicycle, motor cycle, cart, car, bajaj (three wheel small vehicle), solar panel, mill house, electric power or generator, sweeping machine, coffee land, and khat land”.*

1.5. Please indicate how the data on household and other covariables were collected (self/interviewer-administered questionnaire?)

**Response:** Clarified as “Interviewer administered questionnaire was used to collect data on:

1.6. Certain maternal and infant characteristics that have been shown to affect infant growth have not been included. E.g., maternal comorbidities, including HIV status; maternal nutritional status, including obesity; infant birth weight and gestational age. These are important co-variables and if the data are not available, this lack should be included as a limitation.

**Response:** Since we did not have these data collected, now we have indicated this in the limitation section.

1.7. In Table 1, the results of the PHQ-9 scores are presented as means and SD. In Figure 1, the distribution is clearly non-parametric and medians and IQR would be more appropriate. This applies to the distribution of all the continuous variables.
     Other than this, I am not certain that the Figures offer additional benefit.

**Response:** In table one, we provided PHQ-9 in frequency and percentages across the different cut-offs: PHQ score 0, 1-4, 5-9, 10-14, 15-19 and 20-27. We have added the figure to help readers easily understand the distribution of the PHQ-9 data.

1.8. Implications of FINDINGS (typo)

**Response**: corrected as “findings”

1.9. Limitations: Please include the lack of relevant potential confounders and effect modifiers as indicated above.

**Response:** corrected as “Potential confounders and effect modifiers such as maternal HIV/AIDS status, nutritional status, obesity as well as infant birth weight, gestational age and infant morbidities were not included in the study”

1.10. References:
      Please include url and Access Date for on-line references.

**Response:** this is also now corrected

2. **Reviewer #2:**
   2.1. I have annotated the pdf of the manuscript with my comments.

**Response**: Comments from the pdf annotation are all considered and addressed in the main documents. We have used 95% Confidence Interval and p-value to show level of significance.

2.2. General comment: English needs a thorough check as the English is not of a good standard, with many grammatical and some spelling mistakes. I have corrected some as I went along but I would recommend that the manuscript is given to a scientific editor to read through and improve the English.

**Response:** Comment well taken and thorough language editing is done.

2.3. The anthropometrical measurements are meaningless given that the population is "under 6 months" and these measurements vary considerably during this period, hence I would remove them, e.g. lower leg length. The z-scores are acceptable as they correct for age (and sex).

**Response**: We thank you for this comment and it is true that anthropometric values vary greatly in this age group. Considering this, rather than removing lower leg length and other anthropometric indicators from the analysis we have adjusted their analysis for age so that the effect of age is considered in the result.

2.4. The results in the text and table for the statistical analysis are incomplete and lack any mention of statistical significance. An association without a mention of a P-value is not helpful for interpretation and can be misleading to the reader. These need to be included as, without them, the data underlying the findings is incomplete.

**Response:** again now we have used both 95% Confidence Interval and P-value to show level of significance.

3. **Reviewer #3:**
   3.1. It is unclear from the abstract that this was a secondary analysis. This should be clarified so readers understand the limitations.

**Response**: Comment accepted and now indicated in the abstract

3.2. The abstract is very dense and contains too many results making it difficult for the reader to get through. I would suggest editing the abstract to be more succinct and present the main findings of the manuscript, not merely describing every finding.

**Response**: Text in the abstract is now revised accordingly.

3.3. Setting section- Are there other important differences in the two districts that may influence the results? Are the populations similar ethnically and culturally or are there distinct practices that may make them distinct?

**Response**: The population in the two settings are similar in terms of ethnicity, religion and other cultural backgrounds. Edits in the text is included now.

3.4. Discussion- As the authors note, the prevalence of maternal depressive symptoms is much lower than many other published studies. Do the authors have a hypothesis as to why this is the case in the cohort? The PHQ-9 has been validated for use in many settings including Ethiopia; however, this may not translate to every region/ ethnic group. Can the authors speak to the validity of the PHQ-9 among the specific population that they screened? Can we be reassured that the results are valid, especially given the somewhat surprising low prevalence?

**Response**: Yes, the prevalence of maternal depression in this study is low. Although it was not our objective to identify factors contributing to the high/low prevalence of depression in this study, possible justification could be that screening using PHQ-9 in the maternal and child health care setting might need further customisation, contextualisation and other considerations. This has been highlighted in the discussion section as:

*“Several factors such as the setting and context how the data was collected, types of screening tools and strategies used, and the skills, competencies and confidence of data collectors in asking sensitive questions in mental health could have all played a role for the low prevalence of depression and lack of association between MMH problems and poor child nutritional status”.*

3.5. The authors note that the lack of associations with maternal depression and infant nutritional outcomes; however, do the authors believe that they have adequate power in the current analysis given the lower than expected rate of maternal depression?

**Response**: This has already been indicated in the discussion section and now we have indicated this explicitly as;

*“The low prevalence of moderate to severe depression in the current study could have lowered the statistical power to detect significant association between the two problems”*

3.6. The data was collected only between October and January and therefore do not reflect the full seasons/year. MMH may vary seasonally because of external factors, food security, etc. Do the authors believe that this is not a issue and the data is representative?

**Response**: Thank you for this feedback, yes this is an important limitation in this study. So this is now highlighted in the limitation section as

*“The study is a facility based survey conducted in specific season during October –January and therefore the findings may not represent the general population in all seasons of the year”.*

3.7. In the conclusion, I think that it is important to state that "...[MMH] problems are not associate..." in this data set. The lack of findings here does not mean that there is no association. The conclusion should clearly state this.

**Response:** This is now revised in the conclusion both in the abstract and main text.

3.8. Please clarify in the footnote of Table 2 what the bolded values represent. This is not currently clear. Additionally there are some 95% CI values that are bolded without bolding of the beta-coefficient. Ensure that this was intentional.

**Response:** Bolded text are now removed from the table.

.
